# On the Treatment of Tubercular Meningitis

**Published:** 1853-03

**Authors:** O. C. Gibbs

**Affiliations:** Perry, Lake Co., Ohio


*On the Treatment of Tubercular Meningitis.* By O. C. GIBBS, M. D., of Lake Co., Ohio.

The time was, and at no very distant date, when all diseases of the brain, occurring in children, accompanied with, or terminating in, an effusion of fluid into the ventricles of that organ, or between its investing membranes, were denominated Acute Hydrocephalus. And perhaps it would not be too much to say that diseases unaccompanied with that symptom, or without that termination, have been thus classified, irrespective of their pathological conditions.

Dr. Marshall Hall was perhaps the first who divided the so-called acute hydrocephalus into the true and spurious disease, established their non-identity, and proved the diversity of their pathology. The distinction drawn has ever since been practically recognized by the profession.

Recently another division has been made in the disease usually known as hydrocephalus. To whom the honor is due, is to me, immaterial. Dr. West says, "We are led to the conclusion that inflammation of the brain occurs in early life under two different conditions. It now and then comes on in previously healthy children, but occurs much oftener in connection with the tuberculous cachexia, or as the *result* of tubercular deposit in the brain or its membranes." (Lectures on Diseases of Children, p. 58.) But it would seem that he has failed to take practical advantage of the conclusion here expressed; for it appears to me that the occurrence of hydrocephalus in the robust and healthy must depend upon idiopathic meningeal or meningeo-cerebral inflammation; in the scrofulous cachexia the inflammatory action is caused by, and superadded to, the previously existing tubercular disposition. The inflammatory action is usually sthenic in the one case and asthenic in the other. Dr. West is by no means alone in his conclusion, though perhaps he has more distinctly stated it than another writer, whose opinions I have had an opportunity of examining. Dr. Watson says, "The grand predisposing cause of acute hydrocephalus is certainly the scrofulous diathesis, and this is why we see the complaint run so often in families; so that one child having died of that disease, affords much ground for apprehending that others, belonging to the same family, will become victims to it." Yet upon the same page he says: "I need not, I conceive, take any further pains to convince you that the disease is essentially inflammatory. We are inevitably led to that conclusion by the *symptoms*, which nearly resemble those that occur when undoubted inflammation has arisen from injuries of the head; by the *appearances on dissection*, which are *always* such as inflammation may have produced," &c. (Watson's Lectures, p. 270.) Dr. Condie, and others, concur in the opinion expressed in the quotation from Dr. West. Condie says, "Subacute meningitis occurs chiefly in delicate, scrofulous children, especially in those distinguished by great irritability of the brain and nervous system." (Diseases of Children, p. 385.) Again, (p. 390,) he says, "A strong predisposition to the occurrence of chronic meningitis in the *tubercular form*, which, in children, is by far the most frequent, is manifestly hereditary or constitutional." Yet Dr. Condie, in my opinion, correctly denies the sweeping assertion of Dr. Watson, in reference to the post mortem appearances; for he says, "Traces of inflammation in the membranes or substance of the brain, or in other words increased vascularity, or thickening of the membranes, and pseudo-membranes, or purulent effusion, are by no means invariably detected in cases of chronic meningitis." (Diseases of Children, p. 390.) But admitting that the products of inflammation were always present in the brain or its membranes, in cases of death from tubercular meningitis, it certainly would not prove the disease to be essentially and primarily inflammatory; for who does not know, that in cases of death from phthisis, the lungs contain the products of inflammation as invariably as the membranes of the brain in tubercular meningitis. Dr. Simon says, "Tubercle, in itself, is clearly no product of inflammation, but is apt to act as an irritant of surrounding textures, and invite the addition of their inflammatory exudations." (Lectures on Pathology, p. 135.) Hence, we may conclude the disease under consideration to be essentially tubercular, and requiring, in part at least, remedies appropriate for the scrofulous diathesis.

If the above views be correct, it would seem apparent that the remedial means usually resorted to, bloodletting, purgatives, mercurials, &c., are not those best adapted to subdue the disease.

In the country the disease is not of very frequent occurrence. I have seen but four cases, in as many years of medical practice and observation. In these four, one only proved fatal, and in that case, convulsions, hemiplegia and loss of vision occurred before medical advice was taken.

In the three cases of recovery, *Iodide of Potassium* was the remedy depended upon.

The following case, condensed from my note book, is a fair sample of the cases alluded to, and well illustrates the beneficial effects of the iodine treatment.

*August* 11*th*, 1849. I was called to see a female child, aged 17 months. She was the fourth child of scrofulous parents; the two immediately preceding having died of hydrocephalus, at the ages respectively of 28 and 26 months. On the father's side, phthisis was a hereditary disease, on the mother's asthma and cancer. The child was decidedly scrofulous, and presented all the usual symptoms of chronic meningitis in a marked degree. The two preceding children had been treated by a very judicious practitioner; the treatment made use of is unknown to me, but bleeding from the temporal vein was the last resort.

I advised in this case tepid bathing daily; simple, easily digested, but nutritious articles of diet, (which last direction was not attended to,) and such articles as seemed best adapted to remove the derangement of the stomach and bowels to be temporarily resorted to, intermitted and resumed as circumstances might require. The following was ordered and persevered in, with one intermission of two weeks, for about four months.

℞ Iodid. of Potassium gr. xvj.

Sacch. alb. pur. ʒij.

Aquæ ℥ij.

Mix; dose, a teaspoonful once a day, morning.

After two weeks, the patient began slowly to improve, and is now, more than three years since, to all appearances, perfectly well.

In one case, six months after apparent recovery, I was compelled to return to the Iodide of Potassium, which again effected an improvement that has since been permanent.

These cases are, of course, too few to establish the remedial virtues of the Iodide of Potassium in tubercular meningitis; but, if this hastily penned article shall induce others, who have more experience with the disease than myself, to give the remedy a fair trial, and report the result, it will accomplish all I desire.

*Perry, Lake Co., Ohio, February 1st,* 1853.

